# Estimating of the Static and Dynamic Behaviors of Orthogrid-Stiffened FRP Panel Using Reduced-Order Plate Model

**DOI:** 10.3390/ma14174908

**Published:** 2021-08-28

**Authors:** Peng Wang, Yifeng Zhong, Zheng Shi, Dan Luo, Qingshan Yi

**Affiliations:** 1 School of Civil Engineering, Chongqing University, Chongqing 400045, China; 201816131092@cqu.edu.cn (P.W.); 20191601513@cqu.edu.cn (Z.S.); 20171601012@cqu.edu.cn (D.L.); 20154673@cqu.edu.cn (Q.Y.); 2 Key Laboratory of New Technology for Construction of Cities in Mountain Area, Chongqing University, Chongqing 400045, China

**Keywords:** variational asymptotic method, reduced-order plate model, orthogrid-stiffened panel, free-vibration analysis, global buckling

## Abstract

The orthogrid-stiffened FRP panel (OSFP) is a generic structural element in weight-sensitive structure applications. Based on the variational asymptotic method, a 2D reduced-order plate model (2D-RPM) of OSFP was constructed through matching the strain energy of the original panel for static and dynamic analyses. The local field distributions were recovered using the recovery relationship and global response. The relative influences of select parameters on the effective performance of the OSFP were revealed by parametric studies. The comparative results showed that the effective performance of the OSFP predicted by the 2D-RPM were consistent with those predicted by the 3D finite element model, but the computational efficiency was greatly improved. The stiffener height had the greatest influence on the natural frequency of the panel. The layup configurations of laminates had significant influences on the equivalent stiffness and buckling load of the OSFP but had little effect on the vibration modes, which could be varied by adjusting the stiffening forms.

## 1. Introduction

Fiber-reinforced polymer (FRP) has the advantages of being lightweight and having high strength, corrosion resistance, and tailorability. It is a new type of material that exhibits excellent performances, and has been extensively used in civil engineering, shipbuilding, aerospace and so on. Under the same load capacity, the weight of an FRP bridge is only 30% of that of a steel bridge and 5% of that of a reinforced concrete bridge. Due to the excellent properties of FRP, it has gradually become a substitute for traditional building materials (e.g., steel and concrete). Compared with traditional steel and concrete bridges, FRP bridges are not only lightweight but are also more convenient to manufacture, transport, and install. Furthermore, they have long service lives of more than 100 years [1].

The orthogrid-stiffened FRP panel (OSFP) is characterized by a lattice of rigid, interconnected stiffeners, which is a generic structural element in weight-sensitive structure applications [2]. Its stiffness, buckling, and vibration characteristics are not only related to the stiffening forms but are also closely related to the structural and material parameters, which increases the difficulty of analysis. At present, most analysis methods for stiffened panels can be summarized as numerical methods, analytical methods, and a combination of the two methods. The calculation models for the effective analysis of stiffened panels can be divided into finite element models (FEMs) [3,4,5,6,7,8], discrete stiffener models (DSMs) [9], and smeared stiffener models (SSMs) [10]. The stiffeners are modeled as members with axial bending/torsion stiffnesses on the attached skin in DSM, and they can only be effectively applied for the analysis of stiffened panels with thin skins and rigid stiffeners. The geometric features of the stiffeners and skin are contained in FEM, which is more flexible and accurate than DSM. However, it not only requires a long simulation time due to the large computational workload, but also the inability to set different configurations and materials during preliminary design and optimization [11]. The basic idea of SSM is to smear the stiffnesses of the stiffeners into the panel and calculate the effective properties [12,13,14]. This may be applicable to the overall analysis of stiffened panels with intensive interconnected stiffeners but not to the stress and strain analysis of stiffeners [15].

The global buckling is considered as the main failure mode of stiffened panels under axial compression or/and external pressure according to the failure mode map [16,17]. Among the methods used to study the global buckling of stiffened panels, extensive studies have concentrated on the FEM and SSM [18,19]. The extension–bending coupling interaction caused by the eccentricity of the stiffeners was neglected in the work by Chen et al. [20], which resulted in the imprecise buckling prediction results [21]. Abhijit et al. [22] used the FEM to study the free vibration characteristics of stiffened plates with symmetric stiffeners. Later, the isoparametric stiffened-plate element was introduced to analyze the free vibration of eccentrically stiffened plates [23,24]. Lam et al. [25] explored the vibrations of stiffened plates by dividing the plate domain into appropriate rectangular segments.

On the basis of literature review, there were some issues in the existing methods for predicting the effective performance of stiffened panels. First, the shear stiffness matrix cannot be predicted using the homogenized elastic constants and plate thickness together with classic plate theory. Second, due to the assumptions for defining the kinematics, the models were usually applicable to some specific form of stiffeners. Third, few plate models can accurately predict the local stress and strain distributions of stiffeners, which were of great significance to the failure analysis of stiffened panels.

Recently, Yu and Zhong [26,27,28,29] put forward a new multiscale modeling technique to deal with dimensionality-reducing structures based on the variational asymptotic method (VAM) [30]. With this method, the small structural parameters (such as the thickness–width ratio) were used to asymptotically expand the energy functional, and the higher-order terms were removed to obtain the approximate energy of different orders and the corresponding dimension reduction model, which achieved a good tradeoff between accuracy and effectiveness. In this work, a VAM-based reduced-order plate model of stiffened FRP panel was established to solve the three issues mentioned above. The influences of geometric parameters (such as height, thickness, length-width ratio, and period length) on the effective performance of the OSFP were investigated. Finally, the displacements and free-vibration modes of stiffened FRP panels with different stiffening forms, such as the orthogrid-, T- and blade-stiffened panels, were compared. To the author’s knowledge, this method has never been used for this purpose.

## 2. Theoretical Formulation for Reduced-Order Plate Model Using VAM

### 2.1. Kinematics of the OSFP

**Figure 1:**
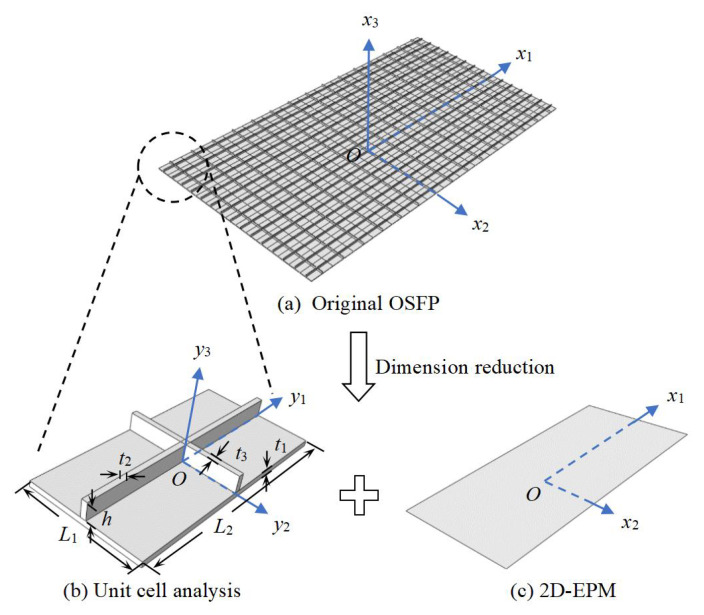
Dimension reduction analysis of the original orthogrid-stiffened FRP panel (OSFP).

As shown in Figure 1, if the sizes of the whole panel (denoted by the macro-coordinates $x_{i}$) are much larger than those of a unit cell (denoted by the micro-coordinates $y_{i}$), then $y_{i}=x_{i}/\xi$ (ξ is a small parameter), and the derivative of the function $f^{\xi }\left(x_{\alpha }\right)$ with respect to $x_{\alpha }$ is

(1) $$\frac{\partial f^{\xi }\left(x_{\alpha }\right)}{\partial x_{\alpha }}={\left.\frac{\partial f\left(x_{\alpha };y_{i}\right)}{\partial x_{\alpha }}\right|}_{y_{i}=\mathrm{const}}+{\left.\frac{1}{\xi }\frac{\partial f\left(x_{\alpha };y_{i}\right)}{\partial y_{i}}\right|}_{x_{\alpha }=\mathrm{const}}\equiv f_{,\alpha }+\frac{1}{\xi }f_{;i}$$

where i,j=1,2,3; α,β=1,2.

To construct a reduced-order plate model of the OSFP using VAM, the 3D displacement field of the original OSFP $u_{i}$ need to be represented by using 2D plate variables $v_{i}$ such as

(2) $$\begin{array}{c} u_{1}\left(x_{\alpha };y_{i}\right)=\underline{v_{1}\left(x_{1},x_{2}\right)-\xi y_{3}v_{3,1}\left(x_{1},x_{2}\right)}+\xi w_{1}\left(x_{\alpha };y_{i}\right)\\ u_{2}\left(x_{\alpha };y_{i}\right)=\underline{v_{2}\left(x_{1},x_{2}\right)-\xi y_{3}v_{3,2}\left(x_{1},x_{2}\right)}+\xi w_{2}\left(x_{\alpha };y_{i}\right)\\ u_{3}\left(x_{\alpha };y_{i}\right)=\underline{v_{3}\left(x_{1},x_{2}\right)}+\xi w_{3}\left(x_{\alpha };y_{i}\right) \end{array}$$

where $w_{i}$ is the fluctuating function to be solved, and the underlined terms should meet the following constraints

(3) $$\begin{array}{c} v_{1}=\langle u_{1}\rangle +\xi \langle y_{3}\rangle v_{3,1}\\ v_{2}=\langle u_{2}\rangle +\xi \langle y_{3}\rangle v_{3,2}\\ v_{3}=\langle u_{3}\rangle \end{array}$$

where 〈·〉 represents the volume integration over the unit cell.

The non-underlined terms should satisfy the following conditions

(4) $$\langle \xi w_{i}\rangle =0$$

The 3D strain field can be expressed as

(5) $$\Gamma_{ij}=\frac{1}{2}\left(\frac{\partial u_{i}}{\partial x_{j}}+\frac{\partial u_{j}}{\partial x_{i}}\right)$$

Plugging Equation (2) into Equation (5) gives the 3D strain field as

(6) $$\begin{array}{c} \Gamma_{11}=\varepsilon_{11}+\xi y_{3}\kappa_{11}+w_{1,1}\\ 2\Gamma_{12}=2\varepsilon_{22}+2\xi y_{3}\kappa_{12}+w_{1,2}+w_{2,1}\\ \Gamma_{22}=\varepsilon_{22}+\xi y_{3}\kappa_{22}+w_{2,2}\\ 2\Gamma_{13}=w_{1,3}+w_{3,1}\\ 2\Gamma_{23}=w_{2,3}+w_{3,2}\\ \Gamma_{33}=w_{3,3} \end{array}$$

where $\varepsilon_{\alpha \beta }$ and $\kappa_{\alpha \beta }$ can be expressed as

(7) $$\begin{array}{c} \varepsilon_{11}\left(x_{1},x_{2}\right)=v_{1,1}\left(x_{1},x_{2}\right),\;\varepsilon_{22}\left(x_{1},x_{2}\right)=v_{2,2}\left(x_{1},x_{2}\right),\\ 2\varepsilon_{12}\left(x_{1},x_{2}\right)=v_{1,2}\left(x_{1},x_{2}\right)+v_{2,1}\left(x_{1},x_{2}\right),\\ \kappa_{11}\left(x_{1},x_{2}\right)=-v_{3,11}\left(x_{1},x_{2}\right),\;\kappa_{22}\left(x_{1},x_{2}\right)=-v_{3,22}\left(x_{1},x_{2}\right),\\ \kappa_{12}\left(x_{1},x_{2}\right)=-v_{3,12}\left(x_{1},x_{2}\right) \end{array}$$

The 3D strain field can be obtained as

(8) $$\begin{array}{c} \Gamma_{e}={\left[\Gamma_{11}\;\Gamma_{22}\;2\Gamma_{12}\right]}^{T}=\varepsilon +x_{3}\kappa +\partial_{e}w_{\left|\right|}\\ 2\Gamma_{s}={\left[2\Gamma_{13}\;2\Gamma_{23}\right]}^{T}=w_{\left|\right|}+\partial_{t}w_{3}\\ \Gamma_{t}=\Gamma_{33}=w_{3,3} \end{array}$$

where $\Gamma_{e},\Gamma_{s},\Gamma_{t}$ are strain matrices of 3D-FEM; ${\left(\right)}_{\left|\right|}={\left[{\left(\right)}_{1}\;{\left(\right)}_{2}\right]}^{T}$, $\varepsilon ={\left[\begin{array}{ccc} \varepsilon_{11} & 2\varepsilon_{12} & \varepsilon_{22} \end{array}\right]}^{T}$, $\kappa ={\left[\kappa_{11}\;\kappa_{12}+\kappa_{21}\;\kappa_{22}\right]}^{T}$, and

(9) $$\partial_{e}=\left[\begin{array}{cc} {\left(\right)}_{,1} & 0\\ {\left(\right)}_{,2} & {\left(\right)}_{,1}\\ 0 & {\left(\right)}_{,2} \end{array}\right],\partial_{t}=\left\{\begin{array}{c} {\left(\right)}_{,1}\\ {\left(\right)}_{,2} \end{array}\right\}$$

**Figure 2:**
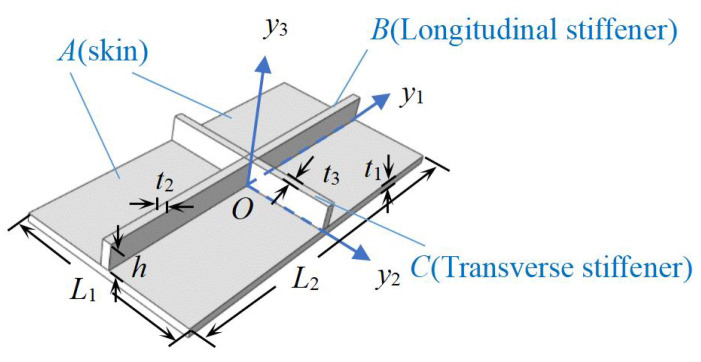
Decomposition diagram of unit cell within the OSFP.

As shown in Figure 2, the unit cell within the OSFP can be divided into three parts to facilitate the integral solution. Then we obtain the strain energy of the panel as

(10) $$U=\frac{1}{2}\int_{-a/2}^{a/2}\int_{-b/2}^{b/2}\frac{1}{\Omega }U_{\Omega }dx_{2}dx_{1}$$

where

(11) $$\begin{array}{c} U_{\Omega }=\int_{-t_{1}}^{0}\int_{-\frac{L_{1}}{2}}^{\frac{L_{1}}{2}}\int_{-\frac{L_{2}}{2}}^{\frac{L_{2}}{2}}\Gamma_{A}^{T}D_{A}\Gamma_{A}dy_{1}dy_{2}dy_{3}\\ \;+\int_{0}^{h}\int_{-\frac{L_{1}}{2}}^{\frac{L_{1}}{2}}\int_{-\frac{t_{2}}{2}}^{\frac{t_{2}}{2}}\Gamma_{B}^{T}D_{B}\Gamma_{B}dy_{1}dy_{2}dy_{3}\\ \;+\int_{0}^{h}\int_{-\frac{t_{3}}{2}}^{\frac{t_{3}}{2}}\int_{-\frac{L_{2}}{2}}^{\frac{L_{2}}{2}}\Gamma_{C}^{T}D_{C}\Gamma_{C}dy_{1}dy_{2}dy_{3} \end{array}$$

with the subscripts *A*, *B*, and *C* representing the skin, longitudinal stiffener, and transverse stiffener, respectively.

Equation (10) can be rewritten as

(12) $$\begin{array}{c} U=\frac{1}{2}\int_{-a/2}^{a/2}\int_{-b/2}^{b/2}\frac{1}{\Omega }\langle \Gamma^{T}D\Gamma \rangle dx_{2}\;dx_{1}\\ \;=\frac{1}{2}\int_{-a/2}^{a/2}\int_{-b/2}^{b/2}\frac{1}{\Omega }\langle {\left\{\begin{array}{c} \Gamma_{e}\\ 2\Gamma_{s}\\ \Gamma_{t} \end{array}\right\}}^{T}\left[\begin{array}{ccc} D_{e} & D_{es} & D_{et}\\ D_{es}^{T} & D_{s} & D_{st}\\ D_{et}^{T} & D_{st}^{T} & D_{t} \end{array}\right]\left\{\begin{array}{c} \Gamma_{e}\\ 2\Gamma_{s}\\ \Gamma_{t} \end{array}\right\}\rangle dx_{2}\;dx_{1} \end{array}$$

where $D_{e},D_{es},D_{et},D_{s},D_{st}$, and $D_{t}$ are the corresponding sub-matrices of three-dimensional 6×6 material matrix.

The virtual work done by the applied loads is

(13) $$\begin{array}{c} E=\int_{-b/2}^{b/2}\int_{-a/2}^{a/2}\frac{1}{\Omega }\left(\langle f_{i}u_{i}\rangle +\beta_{i}u_{i}^{-}+\tau_{i}u_{i}^{+}\right)dx_{1}dx_{2}\\ \;+\int_{-a/2}^{a/2}\int_{-h/2}^{h/2}{\left.\left(\alpha_{i}u_{i}\right)\right|}_{x_{2}=\pm b/2}dx_{3}dx_{1}+\int_{-b/2}^{b/2}\int_{-h/2}^{h/2}{\left.\left(\alpha_{i}u_{i}\right)\right|}_{x_{1}=\pm a/2}dx_{3}dx_{2} \end{array}$$

where $f_{i}$ is the body force, $\alpha_{i}$ is the traction force applied on the lateral surfaces, $\beta_{i}$ and $\tau_{i}$ denote the traction forces on the bottom and top surface, respectively.

Plugging Equation (2) into Equation (13) gives

(14) $$\begin{array}{c} E=\int_{-b/2}^{b/2}\int_{-a/2}^{a/2}\left(p_{i}v_{i}+q_{\alpha }\Phi_{\alpha }\right)dx_{1}dx_{2}\\ \;+\int_{-a/2}^{a/2}\int_{-h/2}^{h/2}{\left.\left(P_{i}v_{i}+Q_{\alpha }\Phi_{\alpha }\right)\right|}_{x_{2}=\pm b/2}dx_{3}dx_{1}\\ \;+\int_{-b/2}^{b/2}\int_{-h/2}^{h/2}{\left.\left(P_{i}v_{i}+Q_{\alpha }\Phi_{\alpha }\right)\right|}_{x_{1}=\pm a/2}dx_{3}dx_{2}+E^{*} \end{array}$$

where $\Phi_{1}=v_{3,2},\Phi_{2}=-v_{3,1}$, and

(15) $$\begin{array}{c} E^{*}=\int_{-b/2}^{b/2}\int_{-a/2}^{a/2}\frac{1}{\Omega }\left(\langle f_{i}w_{i}\rangle +\beta_{i}w_{i}^{-}+\tau_{i}w_{i}^{+}\right)dx_{1}dx_{2}\\ \;+\int_{-a/2}^{a/2}\int_{-h/2}^{h/2}{\left.\left(\alpha_{i}w_{i}\right)\right|}_{x_{2}=\pm b/2}dx_{3}dx_{1}+\int_{-b/2}^{b/2}\int_{-h/2}^{h/2}{\left.\left(\alpha_{i}w_{i}\right)\right|}_{x_{1}=\pm a/2}dx_{3}dx_{2} \end{array}$$

The values of $p_{i}$, $q_{\alpha }$, $P_{i}$, and $Q_{\alpha }$ in Equation (14) can be calculated as

(16) $$\begin{array}{c} p_{i}=\frac{1}{\Omega }\left(\langle f_{i}\rangle +\beta_{i}+\tau_{i}\right)\\ q_{1}=\frac{1}{\Omega }\left(-x_{3}^{-}\beta_{2}-x_{3}^{+}\tau_{2}-\langle x_{3}f_{2}\rangle \right)\\ q_{2}=\frac{1}{\Omega }\left(x_{3}^{-}\beta_{1}+x_{3}^{+}\tau_{1}+\langle x_{3}f_{1}\rangle \right)\\ P_{i}=\langle \langle \alpha_{i}\rangle \rangle \\ Q_{1}=-\langle \langle x_{3}\alpha_{2}\rangle \rangle \\ Q_{2}=\langle \langle x_{3}\alpha_{1}\rangle \rangle \end{array}$$

According to VAM, $E^{*}$ can be ignored, and the total potential energy is

(17) $$\begin{array}{c} \delta \Pi =\delta U-\delta E\\ \;=\int_{-b/2}^{b/2}\int_{-a/2}^{a/2}\left(\frac{1}{2}\delta \langle \Gamma^{T}\bar{D}\Gamma \rangle -p_{i}\delta v_{i}-q_{\alpha }\delta \Phi_{\alpha }\right)dx_{1}dx_{2}\\ \;+\int_{-a/2}^{a/2}\int_{-h/2}^{h/2}{\left.\left(P_{i}\delta v_{i}+Q_{\alpha }\delta \Phi_{\alpha }\right)\right|}_{x_{2}=\pm b/2}dx_{3}dx_{1}\\ \;+\int_{-b/2}^{b/2}\int_{-h/2}^{h/2}{\left.\left(P_{i}\delta v_{i}+Q_{\alpha }\delta \Phi_{\alpha }\right)\right|}_{x_{1}=\pm a/2}dx_{3}dx_{2} \end{array}$$

### 2.2. VAM-Based Reduction Analysis of OSFP

#### 2.2.1. Zeroth-Order Approximation

Plugging Equation (8) into Equation (17) gives the total potential energy density as

(18) $$\begin{array}{c} 2\Pi =\langle \langle \underline{\underline{{\left(\varepsilon +x_{3}\kappa \right)}^{T}D_{e}\left(\varepsilon +x_{3}\kappa \right)}}+\underline{2{\left(\varepsilon +x_{3}\kappa \right)}^{T}D_{e}\partial_{e}w_{||\alpha }}\\ \;+\underline{2{\left(\partial_{e}w_{||\alpha }\right)}^{T}D_{e}\partial_{e}w_{\left|\right|}}+2{\left(\varepsilon +x_{3}\kappa \right)}^{T}D_{es}w_{||,3}+\underline{2{\left(\varepsilon +x_{3}\kappa \right)}^{T}D_{es}\partial_{t}w_{3\alpha }}\\ \;+\underline{2{\left(\partial_{e}w_{||\alpha }\right)}^{T}D_{es}\left(w_{||,3}+\partial_{t}w_{3\alpha }\right)}+2{\left(\varepsilon +x_{3}\kappa \right)}^{T}D_{et}w_{3,3}\\ \;+\underline{2{\left(\partial_{e}w_{||\alpha }\right)}^{T}D_{et}w_{3,3}}+w_{||,3}^{T}D_{s}w_{||,3}+\underline{2w_{||,3}^{T}D_{s}\partial_{t}w_{3\alpha }}+\underline{2{\left(\partial_{t}w_{3,\alpha }\right)}^{T}D_{s}\partial_{t}w_{3,\alpha }}\\ \;+2w_{||,3}^{T}D_{st}w_{3,3}+\underline{2{\left(\partial_{t}w_{3,\alpha }\right)}^{T}D_{st}w_{3,3}+D_{t}w_{3,3}^{2}}\rangle \rangle \\ \;-\underline{2\left(\langle \langle f_{i}^{T}w_{i}\rangle \rangle +\tau_{i}^{T}w_{i}^{T}+\beta_{i}^{T}w_{i}^{T}\right)} \end{array}$$

where the underlined items and the double-underlined item can be ignored according to VAM.

To impose the constraints on the fluctuating function, we introduce the Lagrange multipliers $\lambda_{i}$, such as

(19) $$\delta \left(\Pi +\lambda_{i}\langle w_{i}\rangle \right)=0$$

The zeroth-order approximate variational expression is

(20) $$\langle \begin{array}{c} \left[{\left(\varepsilon +x_{3}\kappa \right)}^{T}D_{es}+w_{||,3}^{T}D_{s}+w_{3,3}^{T}D_{st}^{T}\right]\delta w_{||,3}\\ +\lambda_{i}\delta w_{i}+\left[{\left(\varepsilon +x_{3}\kappa \right)}^{T}D_{et}+w_{||,3}^{T}D_{st}+w_{3,3}^{T}D_{t}\right]\delta w_{3,3} \end{array}\rangle =0$$

The corresponding Euler–Lagrange equations are

(21) $$\begin{array}{c} {\left[{\left(\varepsilon +x_{3}\kappa \right)}^{T}D_{es}+w_{||,3}^{T}D_{s}+w_{3,3}^{T}D_{st}^{T}\right]}_{,3}=\lambda_{\left|\right|}\\ {\left[{\left(\varepsilon +x_{3}\kappa \right)}^{T}D_{et}+w_{||,3}^{T}D_{st}+w_{3,3}^{T}D_{t}\right]}_{,3}=\lambda_{3} \end{array}$$

where $\lambda_{\left|\right|}={\left[\lambda_{1}\;\lambda_{2}\right]}^{T}$.

The boundary conditions of the top and bottom of the panel can be defined as

(22) $$\begin{array}{c} {\left[{\left(\varepsilon +x_{3}\kappa \right)}^{T}D_{es}+w_{||,3}^{T}D_{s}+w_{3,3}D_{st}^{T}\right]}^{+/-}=0\\ {\left[{\left(\varepsilon +x_{3}\kappa \right)}^{T}D_{et}+w_{||,3}^{T}D_{st}+w_{3,3}D_{t}\right]}^{+/-}=0 \end{array}$$

where the superscript “+ / −” indicates the items at the top and bottom of the panel.

From these conditions, we can solve $w_{\left|\right|}$ and $w_{3}$ as

(23) $$w_{\left|\right|}={\langle -\left(\varepsilon +x_{3}\kappa \right){\bar{D}}_{es}D_{s}^{-1}\rangle }^{T},w_{3}=\langle -\left(\varepsilon +x_{3}\kappa \right){\bar{D}}_{et}D_{t}^{-1}\rangle$$

where

(24) $${\bar{D}}_{es}=D_{es}-{\bar{D}}_{et}D_{st}^{T}{\bar{D}}_{t}^{-1},{\bar{D}}_{et}=D_{et}-D_{es}D_{s}^{-1}D_{st},{\bar{D}}_{t}=D_{t}-D_{sl}^{T}D_{s}^{-1}D_{st}$$

Plugging Equation (23) into Equation (18) gives the zeroth-order strain energy as

(25) $$U_{2D}=\frac{1}{2}\langle {\left(\varepsilon +x_{3}\kappa \right)}^{T}{\bar{D}}_{e}\left(\varepsilon +x_{3}\kappa \right)\rangle =\frac{1}{2}{\left\{\begin{array}{c} \varepsilon \\ \kappa \end{array}\right\}}^{T}\left[\begin{array}{cc} A & B\\ B^{T} & D \end{array}\right]\left\{\begin{array}{c} \varepsilon \\ \kappa \end{array}\right\}$$

where A, D and B are tensile, bending, and coupling stiffness sub-matrix, respectively, and can be expressed as

(26) $$\begin{array}{c} A=\langle \langle {\bar{D}}_{e}\rangle \rangle ,B=\langle \langle x_{3}{\bar{D}}_{e}\rangle \rangle ,D=\langle \langle x_{3}^{2}{\bar{D}}_{e}\rangle \rangle ,\\ {\bar{D}}_{e}=D_{e}-{\bar{D}}_{es}D_{s}^{-1}D_{es}^{T}-{\bar{D}}_{et}D_{et}^{T}/{\bar{D}}_{t} \end{array}$$

#### 2.2.2. Transforming into Reissner–Mindlin Model

There are two additional transverse shear strains $\gamma ={\left\lfloor 2\gamma_{13}\;2\gamma_{23}\right\rfloor }^{T}$ in the Reissner–Mindlin model. To transform Equation (25) into the Reissner–Mindlin model, we must eliminate the coupled stiffness terms between ϵ and γ as follows:

(27) $$\begin{array}{c} 2\Pi_{\Omega }=\Gamma^{T}{\bar{D}}_{e}\Gamma =R^{T}AR+2R^{T}B\gamma +\gamma^{T}C\gamma \\ \;=R^{T}\left(A-{BC}^{-1}B^{T}\right)R+{\left(\gamma +C^{-1}B^{T}R\right)}^{T}C\left(\gamma +C^{-1}B^{T}R\right) \end{array}$$

where R is Reissner–Mindlin generalized strains.

The final form of the total energy can be expressed as

(28) $$2\Pi_{R}=R^{T}XR+\gamma^{T}G\gamma +2R^{T}F$$

where F is a load-related term and

(29) $$\begin{array}{c} X=A-BC^{-1}B^{T}\\ G=C \end{array}$$

The resultant stress of the panel can be expressed as

(30) $$\begin{array}{c} \frac{\partial \Pi_{R}}{\partial \varepsilon_{11}}=N_{11},\;\frac{\partial \Pi_{R}}{\partial 2\varepsilon_{12}}=N_{12},\;\frac{\partial \Pi_{R}}{\partial \varepsilon_{22}}=N_{22}\\ \frac{\partial \Pi_{R}}{\partial \kappa_{11}}=M_{11},\;\frac{\partial \Pi_{R}}{\partial 2\kappa_{12}}=M_{12},\;\frac{\partial \Pi_{R}}{\partial \kappa_{22}}=M_{22}\\ \frac{\partial \Pi_{R}}{\partial 2\gamma_{13}}=Q_{1},\;\frac{\partial \Pi_{R}}{\partial 2\gamma_{23}}=Q_{2} \end{array}$$

Due to the symmetry of the axis and plane, some stiffness components disappear, and the constitutive relation of the OSFP can be obtained as

(31) $$\left(\begin{array}{c} N_{11}\\ N_{22}\\ N_{12}\\ M_{11}\\ M_{22}\\ M_{12}\\ Q_{1}\\ Q_{2} \end{array}\right)=\left(\begin{array}{cccccccc} A_{11} & A_{12} & 0 & 0 & 0 & 0 & 0 & 0\\ A_{12} & A_{22} & 0 & 0 & 0 & 0 & 0 & 0\\ 0 & 0 & A_{66} & 0 & 0 & 0 & 0 & 0\\ 0 & 0 & 0 & D_{11} & D_{12} & 0 & 0 & 0\\ 0 & 0 & 0 & D_{12} & D_{22} & 0 & 0 & 0\\ 0 & 0 & 0 & 0 & 0 & D_{66} & 0 & 0\\ 0 & 0 & 0 & 0 & 0 & 0 & C_{11} & 0\\ 0 & 0 & 0 & 0 & 0 & 0 & 0 & C_{22} \end{array}\right)\left(\begin{array}{c} \varepsilon_{11}\\ \varepsilon_{22}\\ 2\varepsilon_{12}\\ \kappa_{11}\\ \kappa_{22}\\ 2\kappa_{12}\\ \gamma_{13}\\ \gamma_{23} \end{array}\right)$$

The original 3D geometric nonlinear problem in Equation (17) is mathematically decomposed into constitutive modeling over the unit cell in Equation (31) and geometric nonlinear plate analysis. That is to say, as an alternative to the direct numerical simulation using 3D nonlinear finite element analysis, the global analysis of the OSFP can be reduced to 2D plate analysis using the linear solver in ABAQUS, with the constitutive relation obtained from the constitutive modeling of the unit cell.

### 2.3. Equivalent Density of 2D-RPM

For heterogeneous materials and structures, the equivalent density of the unit cell is usually used to characterize the weight of the reduced-order plate model. The total mass of the unit cell within the OSFP can be expressed as

(32) $$m=m_{1}+m_{2}+m_{3}=\rho_{1}\cdot L_{1}L_{2}\cdot t_{1}+\rho_{2}\cdot L_{2}h\cdot t_{2}+\rho_{3}\cdot L_{1}h\cdot t_{3}$$

where $\rho_{1},\rho_{2}$, and $\rho_{3}$ are the density of the skin, the longitudinal stiffener and transverse stiffener, respectively.

The total volume of the unit cell is

(33) $$V=L_{1}L_{2}\cdot \left(t_{1}+h\right)$$

According to the equivalent density formula, we obtain

(34) $$\rho =\frac{m}{V}$$

The equivalent density of the 2D-RPM can be expressed as

(35) $$\rho^{*}=\frac{\rho_{1}\cdot L_{1}L_{2}\cdot t_{1}+\rho_{2}\cdot L_{2}h\cdot t_{2}+\rho_{3}\cdot L_{1}h\cdot t_{3}}{L_{1}L_{2}\cdot \left(t_{1}+h\right)}$$

## 3. Validation Example

**Figure 3:**
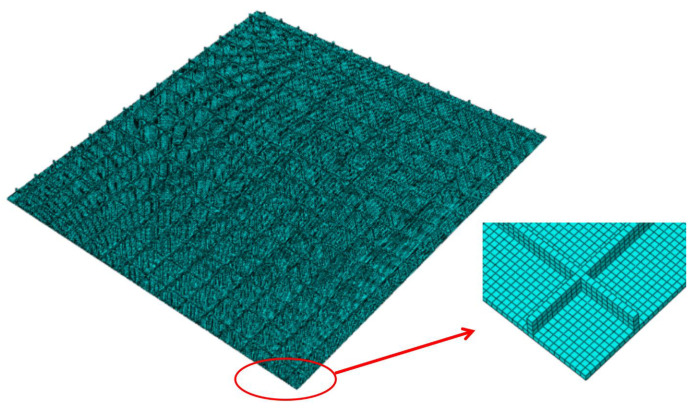
Meshing of 3D finite element model (3D-FEM).

**Figure 4:**
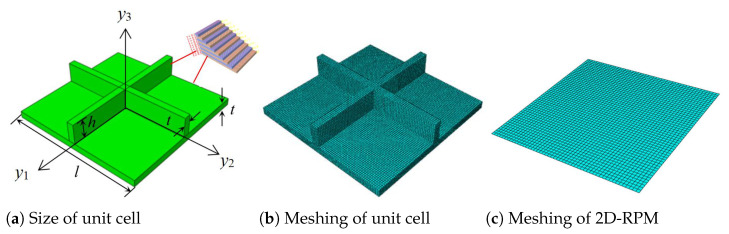
Meshing of unit cell and 2D reduced-order plate model (2D-RPM) of OSFP.

To verify the accuracy and effectiveness of the present reduced model, the static and dynamic behaviors of OSFP predicted by the present model were compared with those of 3D-FEM. The 3D-FEM had 15 unit cells in the $x_{1}$ and $x_{2}$ direction as shown in Figure 3. The equivalent stiffness of OSFP was obtained by variational asymptotic analyzing over the unit cell shown in Figure 4b and inputted into the 2D-RPM (300 mm × 300 mm) using shell elements, as shown in Figure 4c, to analyze the static and dynamic behavior under different boundary conditions. The relative error between the 2D-RPM and 3D-FEM is defined as Error$\;=\frac{|2D-\mathrm{RPM}\;\mathrm{results}-3D-\mathrm{FEM}\;\mathrm{results}|}{3D-\mathrm{FEM}\;\mathrm{results}}\times 100\%$.

**Figure 5:**
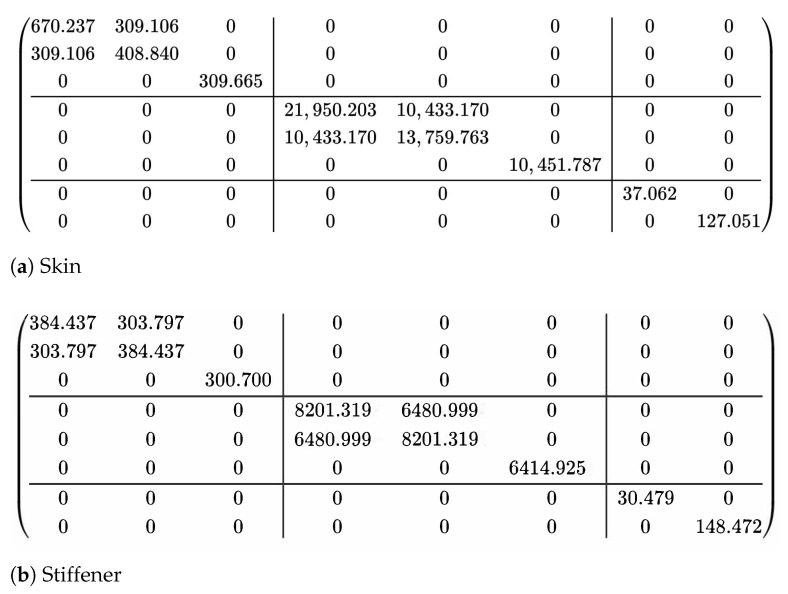
Effective plate properties of the skin and the stiffener calculated by the present model (unit: SI).

The structural parameters shown in Figure 4a were: *l* = 20 mm, *h* = 3 mm, and *t* = 1 mm. The OSFP is made of T300/7901 carbon/epoxy laminates. The layup configuration of skin was ${\left[45/-45/0/-45/45\right]}_{2s}$, and that of stiffener was ${\left[45/-45\right]}_{4s}$. The lamina properties were: $E_{11}=71.76$ GPa, $E_{22}=E_{33}=7.81$ GPa, $G_{12}=G_{13}=2.52$ GPa, $G_{23}=2.11$ GPa, $v_{12}=v_{13}=0.343$, $v_{23}=0.532$, $\rho =1.42\;g/{\mathrm{cm}}^{3}$. The effective plate properties of the skin and the stiffener obtained by present model were given in Figure 5 for reference.

### 3.1. Static Displacement Analysis

**Figure 6:**
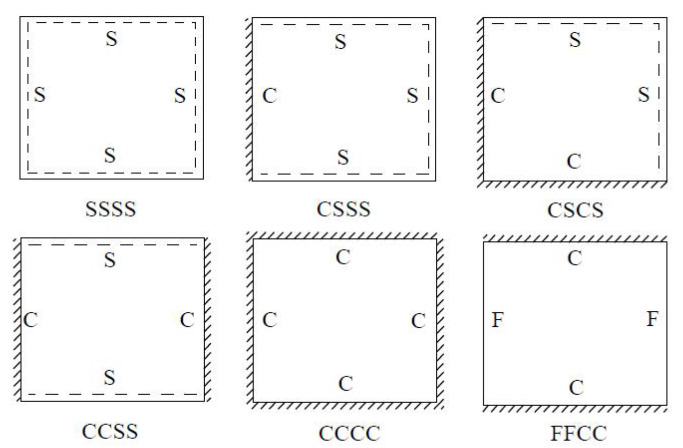
Typical boundary conditions of the OSFP.

Six typical boundary conditions shown in Figure 6, including CCCC, CCSS and CSCS, CSSS, SSSS and FFCC, were used for static displacement analysis. The naming convention of boundary conditions is four letters, where S denotes simply supported constraint, C for fixed constraint, and F for free constraint.

**Figure 7:**
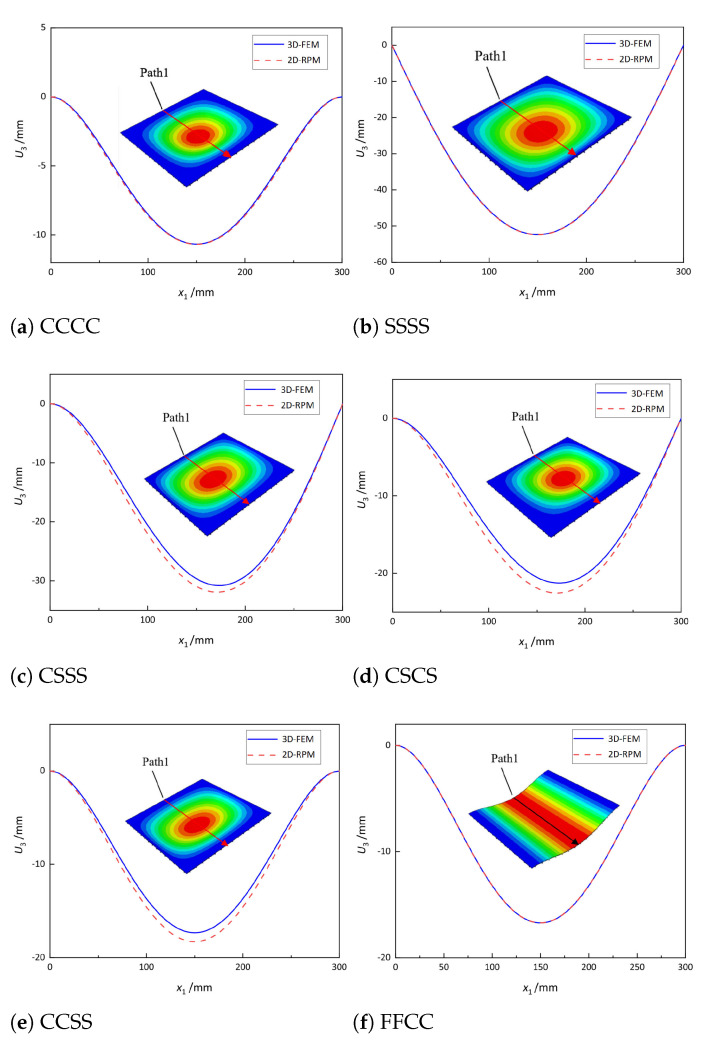
Vertical displacement along Path 1 of the plate under a uniform load of 5 kPa and different boundary conditions.

To verify the effectiveness of the 2D-RPM, a uniform load of 5 kPa was applied to the top surface of the OSFP, and the displacement distributions along Path 1 of the 3D-FEM and 2D-RPM were compared in Figure 7. The comparative results show that the displacement distributions predicted by the 2D-RPM were basically in agreement with those of the 3D-FEM. The differences were due to the different meshing methods used in the two models. The maximum displacement error under the CCSS boundary condition was the largest, but it was still within 5%. It is worth noting that the differences between the 2D-RPM and 3D-FEM in Figure 7c–e were much greater than other cases, which may be due to the gradual enhancement of boundary constraints from SSSS to CCCC. It was concluded that the 2D-RPM can predict the static displacement of the stiffened FRP panel with high accuracy and effectiveness, and the equivalent stiffness obtained from the VAM had sufficient accuracy.

### 3.2. Local Displacement and Stress Field

**Figure 8:**
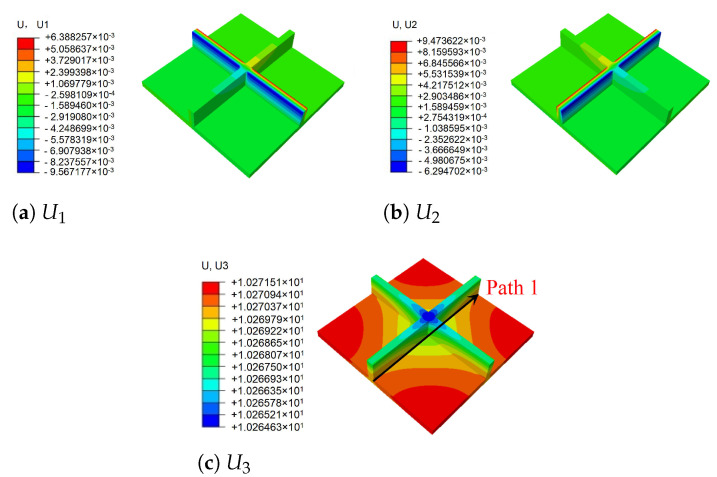
Local displacement fields within the unit cell at the geometric center of the plate (unit: mm).

According to Equation (2), the local displacement distributions within the unit cell at the geometric center of the 2D-RPM can be recovered as shown in Figure 8. It can be observed that the maximum and minimum values of $U_{1}$ and $U_{2}$ were located on opposite sides of the stiffener, and the overall displacement presented a centrosymmetric distribution trend. The smallest value of $U_{3}$ was located at the center of the stiffener, while the maximum value of $U_{3}$ was on the skin. The displacement distribution was centrally symmetric about the intersection point of the stiffeners. The maximum value of $U_{3}$ within the unit cell was 10.27 mm, and the error was about 1% compared with that of 3D-FEM presented in Section 3.1 ($U_{3}$ = 10.17 mm), indicating that the recovered displacement distribution is accurate and can be used to evaluate the location of maximum local displacement.

**Figure 9:**
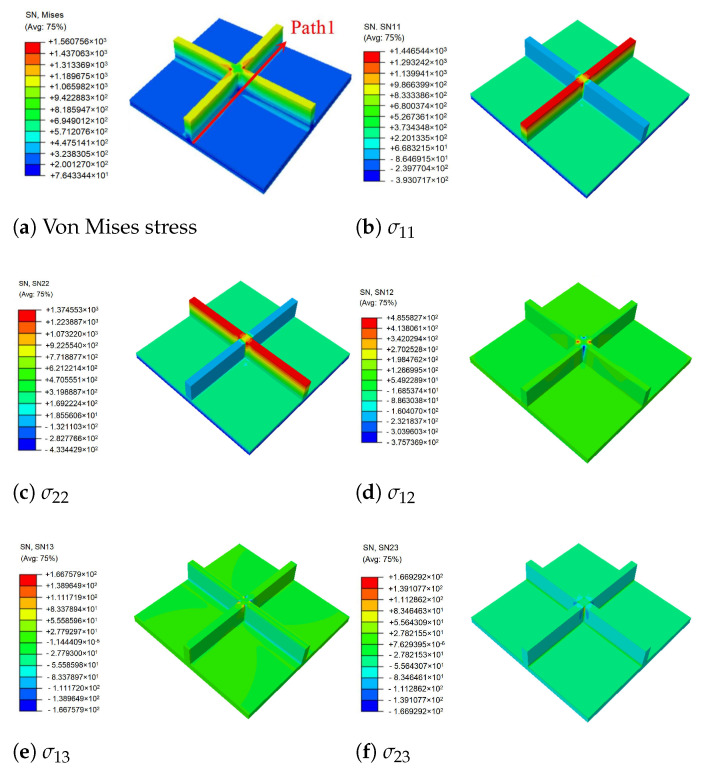
Local stress fields within the unit cell at the geometric center of the plate (unit: MPa).

Figure 9 shows the local stress fields within the unit cell at the geometric center of the plate recovered from Equation (8) and the 3D Hooke’s law. It can be observed that the stiffeners played an important role in the process of load transfer, and there was a large stress concentration at the intersection of the stiffeners and the skin, showing a significant skin-stiffener effect. The stress distribution on the skin was relatively uniform, and there was no evident mutation. It was concluded that the stiffeners improved the bearing capacity of the panel, and the OSFP had a low weight and high strength compared to the ordinary panel.

**Figure 10:**
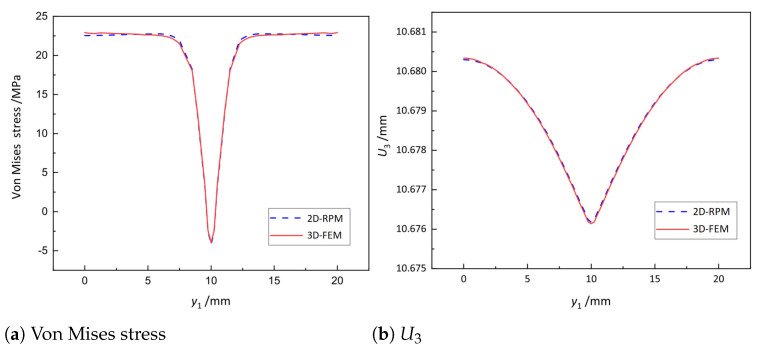
Comparison of von Mises stress and $U_{3}$ distribution along Path 1 within the unit cell at the geometric center of the OSFP.

Figure 10 shows the local von Mises stress and displacement distribution along Path 1 of the skin within the unit cell (as shown in Figure 8c and Figure 9a) predicted by 2D-RPM and 3D-FEM. It can be observed that the local stress and displacement curves predicted by 2D-RPM and 3D-FEM agreed well, and the maximum error was less than 5%. The local stress at the junctions between the skin and the stiffeners decreased significantly, indicating that these regions were very incidental to be damaged.

### 3.3. Free-Vibration Analysis

Table 1 shows the the first four vibration modes and natural frequencies of the OSFP under the CCCC boundary condition predicted by 2D-RPM and 3D-FEM. The vibration modes of the 3D-FEM and 2D-RPM were in good agreement. For example, there were one and two half-waves along the $x_{1}$ direction, two half-waves along the $x_{2}$ direction, and two half-waves along the $x_{1}$ and $x_{2}$ directions for the first, second, third, and forth mode shapes, respectively, for both the 2D-RPM and 3D-FEM results. The natural frequencies of the 3D-FEM and 2D-RPM were also highly consistent, and the maximum error of the natural frequency was less than 6.83%. It was worth noting that the first-order vibration frequency showed relative big error compared with the third and fourth vibration frequencies, which may be because the first-order frequency was more sensitive to different meshing between 2D-RPM and 3D-FEM. The 2D-RPM was more time-efficient than the 3D-FEM in analyzing the vibration modes: the 2D-RPM required 30 s with one CPU as opposed to the nearly 20 min required for the 3D-FEM with four CPUs.

It can be concluded that the 2D-RPM had high accuracy in free-vibration analysis of OSFP under CCCC boundary condition. To further verify the effectiveness of 2D-FEM in analyzing vibration modes, the vibration modes of OSFP under different boundary conditions are analyzed as shown in Table 2. The 3D-FEM and VAM-based 2D-RPM had good consistency in the prediction of natural frequencies and vibration modes under various boundary conditions, and the maximum error of natural frequency was less than 6% under the CCSS boundary condition. The stronger the boundary condition is, the higher the natural frequency is. The natural frequency under CCCC boundary condition was about twice that under SSSS boundary condition. The natural frequencies under CCSS and CSCS boundary conditions were almost the same, but the asymmetry of CSCS boundary condition led to the asymmetry of vibration mode.

### 3.4. Global Buckling Analysis

**Figure 11:**
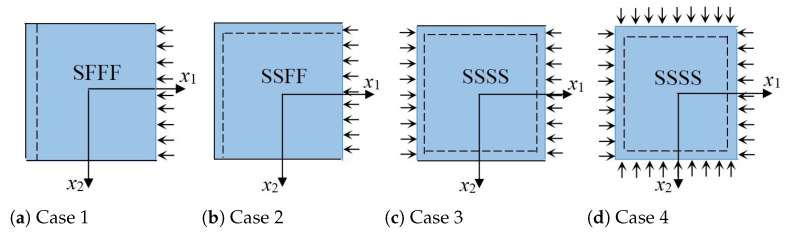
Four combination of boundary and load conditions used in buckling analysis.

To verify the accuracy and effectiveness of 2D-RPM, the buckling modes and critical loads of OSFP under different boundary and load conditions illustrated in Figure 11 are listed in Table 3. The combinations of boundary and load conditions included SFFF/uniaxial (Case 1), SSFF/uniaxial (Case 2), SSSS/uniaxial (Case 3), and SSSS/biaxial (Case 4).

Table 3 shows that the buckling load in Case 3 (471.83 N) was about 9 times that in Case 2 (57.96 N) and 1.577 times that in Case 4 (300.67 N). The buckling loads in Case 1 and Case 2 were basically the same. The error of the critical buckling load under various boundary conditions was less than 5%, indicating that the VAM-based 2D-RPM and 3D-FEM predictions of the global buckling of OSFP agreed closely.

## 4. Parameter Study

The 2D-RPM was selected to conduct parametric study. The material properties and layup configurations of the skin and the stiffeners were the same as those in Section 3, except where explicitly indicated. The boundary conditions for uniaxial buckling analysis were SFFF in Case 1, while the boundary conditions for the free-vibration analysis were CCCC.

### 4.1. Influence of Structural Parameters on Equivalent Plate Properties

**Figure 12:**
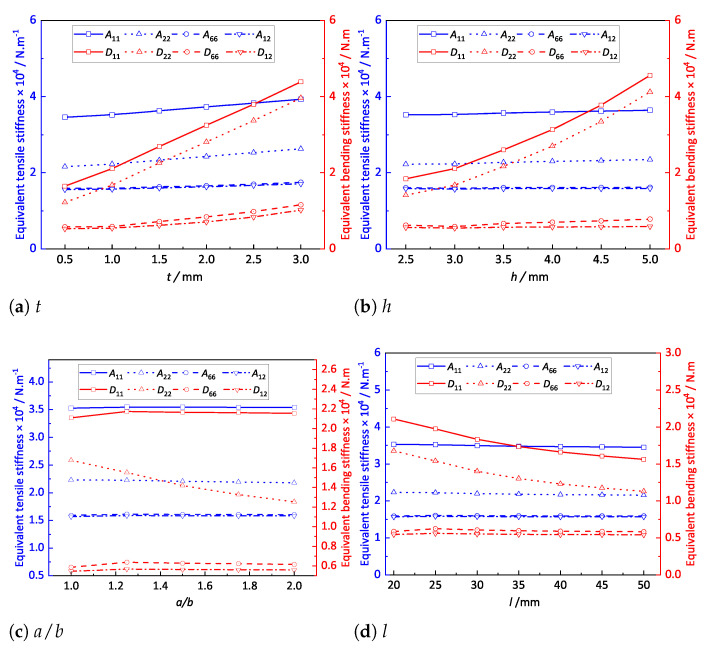
Effects of the structural parameters on the equivalent plate properties of the OSFP.

Figure 12a shows the effect of the stiffener thickness on the equivalent stiffness of the OSFP when the other parameters remained unchanged. The equivalent stiffness $A_{ij}$ and $D_{ij}$ increased with increasing stiffener thickness, and in particular, the bending stiffness components $D_{11}$ and $D_{22}$ increased significantly. This may be because $A_{ij}$ was directly proportional to the cross-sectional area, which increased with increasing stiffener thickness. In contrast, $D_{ij}$ was proportional to the moment of inertia, which was linearly related to the stiffener thickness.

Figure 12b shows the effect of stiffener height on the equivalent stiffness when other parameters remain unchanged. It can be seen that $A_{ij}$ increased linearly and $D_{ij}$ increased nonlinearly with the increasing stiffener height. The reason was that $A_{ij}$ was proportional to the sectional area, which was linear with the stiffener height, while $D_{ij}$ was proportional to the moment of inertia, resulting in a parabolic growth trend.

Figure 12c shows the effect of length–width ratio on the equivalent stiffness. It can be observed that $A_{11}$ and $D_{11}$ had no obvious change with the increasing length–width ratio, while $D_{22}$ decreased significantly. The reason was that the extension area and moment of inertia in $x_{1}$ direction remained unchanged, while the extension area and moment of inertia in the $x_{2}$ direction gradually decreased with the increasing length–width ratio, which led to the nonlinear decrease in equivalent bending stiffness.

Figure 12d shows the effect of the periodic length on the equivalent stiffness of the stiffened FRP panel. It can be observed that $A_{ij}$ and $D_{ij}$ decreased nonlinearly with the increase in periodic length. This was because $A_{ij}$ and $D_{ij}$ were, respectively, proportional to the extension area and the moment inertia, and there was a negative nonlinear relationship between the extension area/moment inertia and the periodic length, resulting in a parabolic downward trend with the increasing periodic length.

### 4.2. Influence of Structural Parameters on Buckling Loads and Natural Frequencies

**Figure 13:**
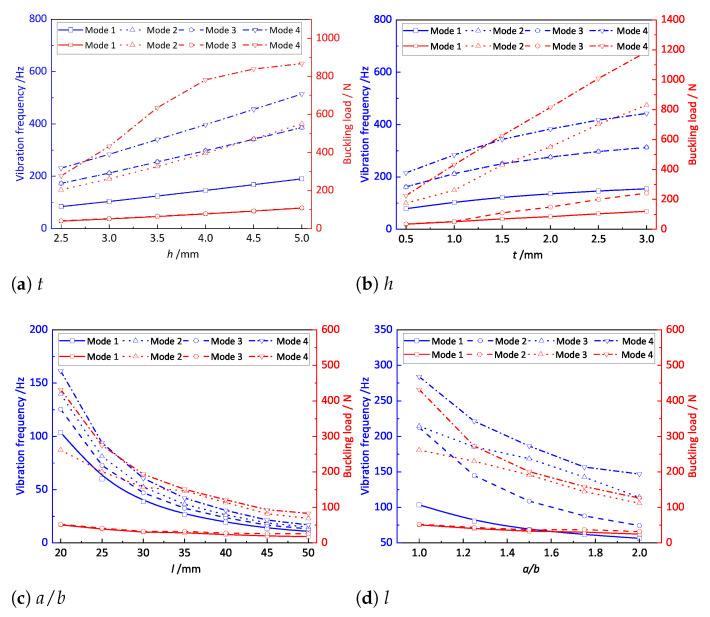
Effects of the structural parameters on the natural frequency and buckling load of the OSFP.

To further investigate the influence of structural parameters on the effective performance of OSFP, the first four buckling loads and natural frequencies of OSFP with different stiffener height, thickness, periodic length, and length–width ratio were calculated by using 2D-RPM, as shown in Figure 13.

The first four natural frequencies of the OSFP increased with increasing stiffener thickness and height and decreased with increasing length–width ratio and periodic length. The effect of the stiffener height *h* on the natural frequency was much greater than that of other structural parameters. The reason is that the variation trends of the equivalent stiffness and equivalent mass were consistent with those of structural parameters, and their influences on the natural frequency might counteract each other. However, the effect of the stiffener height *h* on the equivalent stiffness was much greater than that on the equivalent mass. The buckling load of the OSFP increased with the increase in stiffener thickness and height but decreased with increasing length–width ratio and periodic length, which was the same as the change trend of equivalent stiffness.

### 4.3. Influence of Layup Configuration on the Effective Performance of OSFP

The layup configurations of the laminates would affect the effective performance of the OSFP due to the anisotropy and heterogeneity. In this section, the influences of the layup configuration on the equivalent stiffness, free vibrations, and buckling mode of the OSFP are analyzed. The layup configuration was set to ${\left[0/\theta /0/\theta /0\right]}_{s}$, where θ increased from $0^{\circ }$ to $90^{\circ }$ at $15^{\circ }$ intervals. The boundary condition was fixed on one side and simply supported on three sides (CSSS).

**Figure 14:**
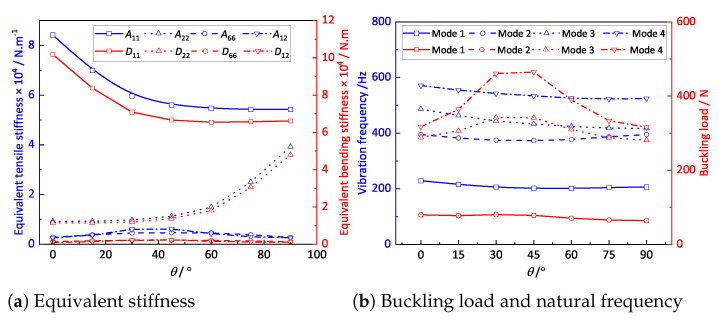
Effects of the layup configuration on the effective performances of the OSFP.

Figure 14a shows the effect of the layup configuration on the equivalent stiffness of the OSFP. With the gradual increase in the ply angle, the stiffness components $A_{11}$ and $D_{11}$ showed nonlinear downward trends, while $A_{22}$ and $D_{22}$ showed significant nonlinear increases when the ply angle was greater than $45^{\circ }$. Figure 14b shows the effect of the layup configuration on the first four natural frequencies and buckling loads of the OSFP. It can be observed that the layup configuration had little effect on the natural frequency, and the first natural frequency first decreased and then increased with the increasing ply angle, reaching the minimum value in the range of 30–$60^{\circ }$ ply angle. The buckling load first increased and then decreased with the increasing ply angle, and the buckling load of each order reached the maximum value at 30–$45^{\circ }$ ply angle.

### 4.4. Influence of $0^{\circ }$-Ply Ratio on the Effective Performance of OSFP

**Figure 15:**
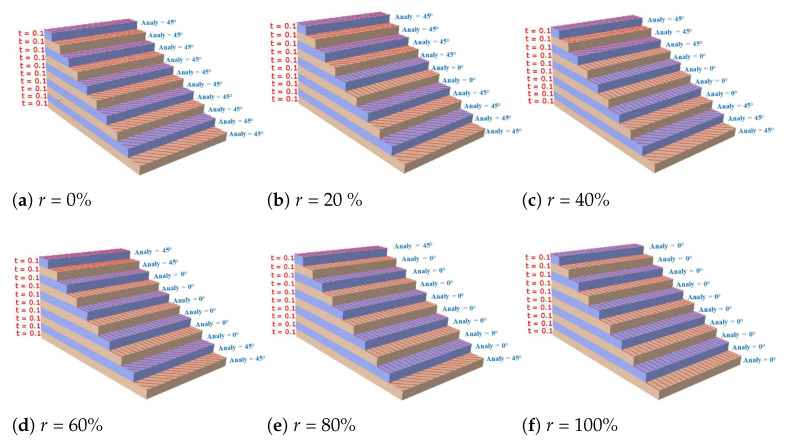
Six different $0^{\circ }$-ply ratios in the layup combination of $0^{\circ }$ and $45^{\circ }$ plies.

The $0^{\circ }$ ply ratio *r* was the ratio of the number of $0^{\circ }$-ply to the total number of ply. To study the influence of $0^{\circ }$-ply ratio on the effective performance of the panel, the 10-layered laminate with combination of $0^{\circ }$ and $45^{\circ }$ plies was considered, and the six layup configurations are illustrated in Figure 15.

**Figure 16:**
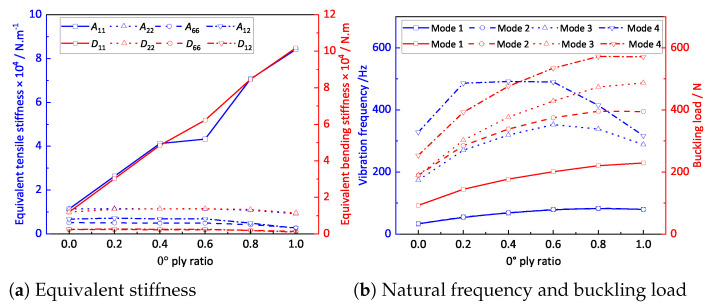
Effects of the structural parameters on the effective performances of the OSFP.

Figure 16a shows that the influences of the $0^{\circ }$-ply ratio on $A_{11}$ and $D_{11}$ were much greater than those on the other stiffness components because the stiffness along the fiber direction ($0^{\circ }$ direction) was stronger. Figure 16b shows that the first natural frequency increased with increasing $0^{\circ }$-ply ratio and reached a maximum value when the $0^{\circ }$ ply ratio was 100%. The third to fourth natural frequencies increased first and then decreased and reached the maximum value when the $0^{\circ }$-ply ratio was between 0.2 and 0.6. With the increase in the $0^{\circ }$-ply ratio, the first to fourth buckling loads of the OSFP increased gradually and reached a maximum value when the $0^{\circ }$ ply ratio was 100%. In engineering applications, the effective performance of the OSFP in the corresponding direction can be improved by adjusting the $0^{\circ }$-ply ratio.

## 5. Comparison with Other Stiffened FRP Panels with Different Stiffening Forms

**Figure 17:**
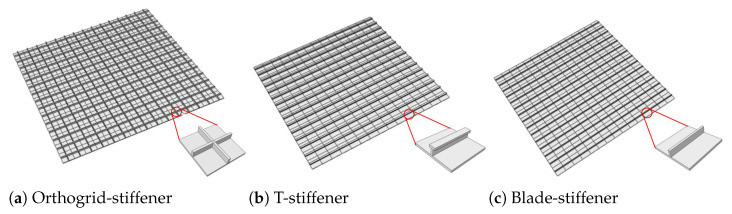
3D finite element model and its unit cell of stiffened FRP panels with different stiffening forms.

To compare the effects of different stiffening forms on the effective performance of the OSFP, the 3D FE models and 2D reduced-order plate models of orthogrid-, T-, and blade-stiffened FRP panel were established. The 3D FE models were obtained by repeating the unit cell 15 times in the $x_{1}$ and $x_{2}$ directions as shown in Figure 17. The structural parameters of unit cell were *l* = 20 mm, *h* = 3 mm, and *t* = 1 mm. The material parameters were the same as in Section 4, and the layup configurations of skin and stiffener were ${\left[45/-45/0/-45/45\right]}_{2s}$ and ${\left[45/-45\right]}_{4s}$, respectively.

**Figure 18:**
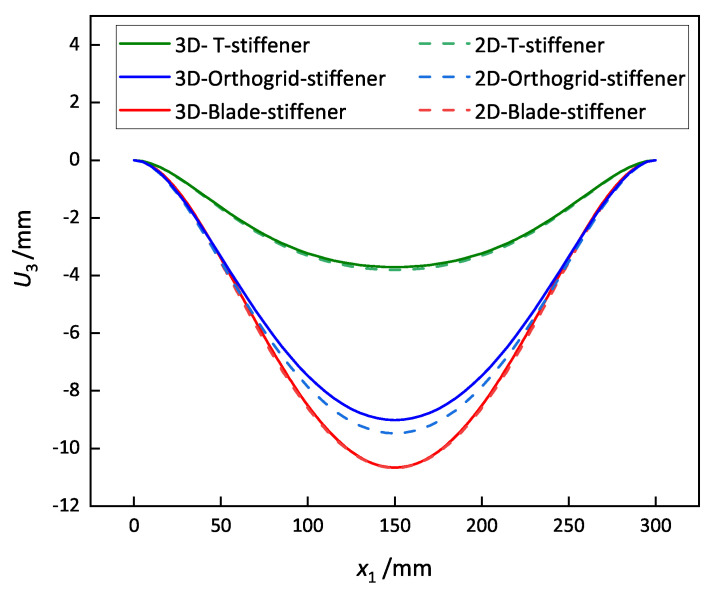
Comparison of the displacements along the center line of the stiffened FRP panel with different stiffening forms under the CCCC boundary condition and a 5 kPa uniform load.

The static displacements along the center line of the stiffened FRP panels under the CCCC boundary condition and 5 kPa of uniform load were analyzed. The comparative results in Figure 18 show that the displacement of the blade-stiffened FRP panel was the largest, followed by the orthogrid- and T-stiffened FRP panels due to the fact that the equivalent bending stiffness of T-stiffened FRP panel was greater than the other two stiffened FRP panels.

Table 4 shows the first four natural frequencies of the stiffened FRP panel with different stiffening forms under the CCCC boundary condition. The first natural frequency of the T-stiffened FRP panel was the largest. From the second order, the natural frequency of the orthogrid-stiffened FRP panel was the largest, followed by the T- and blade-stiffened FRP panels.

The comparative results showed that the natural frequencies of the OSFP increased faster with increasing modal order, while those of the T- and blade-stiffened FRP panels showed little change. The vibration modes of the T- and blade-stiffened FRP panels were basically the same, but the vibration modes of the orthogrid-stiffened FRP panel were very different (there were one and two half-waves along the $x_{1}$ direction, two half-waves along the $x_{2}$ direction, and two half-waves along the $x_{1}$, and $x_{2}$ directions for the first, second, third, and forth mode shapes, respectively). It was concluded that the vibration modes of the stiffened FRP panel could be changed by adjusting the stiffening forms.

## 6. Conclusions

In this work, a VAM-based reduced-order plate model was established to analyze the effective performance of the orthogrid-stiffened FRP panel (OSFP). The influences of the material and structural parameters of the OSFP were investigated by parametric studies, and the following conclusions were drawn.

(1) The results of static displacement, local field distributions, natural frequencies and buckling loads predicted by 2D-RPM were consistent with those of 3D FE model, but the computational efficiency was greatly improved, which verifies the accuracy and effectiveness of the VAM-based reduced-order plate model.

(2) The equivalent stiffness increased gradually with increasing stiffener thickness or height and decreasing length–width ratio or periodic length. The influences of the structural parameters on the buckling load were closely related to the equivalent stiffness. The effect of the stiffener height on the natural frequency of the OSFP was much greater than those of the other structural parameters.

(3) Different layup configurations had significant influences on the equivalent stiffness and buckling load of the OSFP, while they had little effect on the vibration modes. With the increase in the $0^{\circ }$ ply ratio, the equivalent stiffness in the fiber direction increased significantly, and the first buckling load and natural frequency increased gradually. The static displacement and vibration modes of the orthogrid-stiffened FRP panel were different from those of the blade- and T-stiffened FRP panels, indicating that the vibration modes of the stiffened FRP panel could be varied by adjusting the stiffening forms. 

## Figures and Tables

**Table 1:** Comparison of the first four vibration modes and eigenvalues (Hz) of the OSFP predicted by 3D-FEM and 2D-RPM under the CCCC boundary condition.

Order	1	2	3	4
2D-RPM	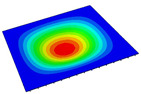 103.68	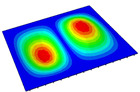 212.48	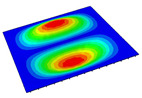 215.68	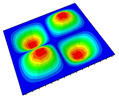 283.97
3D-FEM	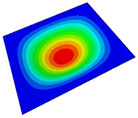 110.76	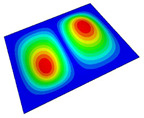 211.62	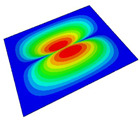 216.33	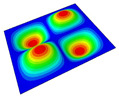 290.39
Error	6.83%	0.41%	0.30%	2.25%

**Table 2:** Comparison of vibration modes and natural frequencies (Hz) of the OSFP under different boundary conditions (BCs).

BCs	SSSS	CSSS	CSCS	CCSS	FFCC
2D-RPM	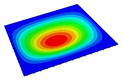 45.48	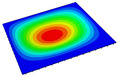 60.11	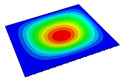 71.43	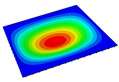 80.29	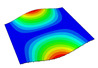 71.93
3D-FEM	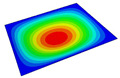 47.56	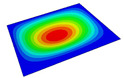 63.15	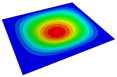 72.37	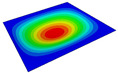 84.98	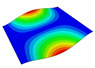 73.37
Error	4.57%	5.06%	1.31%	5.84%	2.00%

**Table 3:** Comparison of global buckling modes and loads (N) predicted by the 2D-RPM and 3D-FEM under different boundary and load conditions.

Order	1	2	3	4
2D-RPM	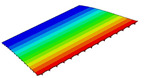 50.97	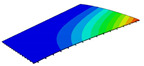 57.96	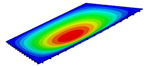 471.83	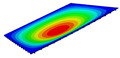 300.67
3D-FEM	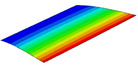 51.24	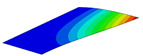 59.27	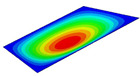 482.12	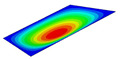 310.42
Error	0.53%	2.26%	2.18%	3.24%

**Table 4:** Influence of the different stiffening forms on the natural frequencies (Hz) of stiffened FRP panels under the CCCC boundary condition.

Orders	T-Stiffened Panel	Orthogrid-Stiffened Panel	Blade-Stiffened Panel
1	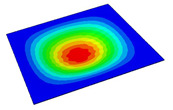 145.88	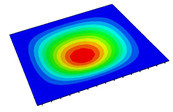 103.68	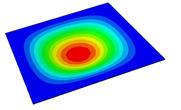 76.24
2	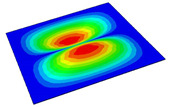 147.50	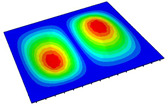 212.48	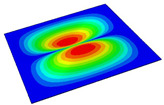 77.54
3	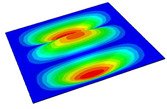 150.75	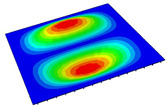 212.48	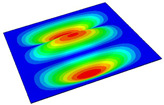 80.68
4	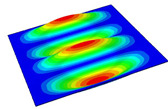 154.38	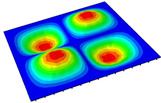 283.97	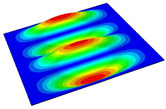 86.30

## Data Availability

Data available on request due to restrictions, e.g., privacy or ethical. The data presented in this study are available on request from the corresponding author. The data are not publicly available due to subsequent analyzes and publications.
